# Supplementary material for: Mapping imported malaria in Bangladesh using parasite genetic and human mobility data
Source: eLife. 2019 Apr 2;8:e43481. doi: 10.7554/eLife.43481 (PMC6478433; doi:10.7554/eLife.43481)
Supplement: Supplementary file 2. [file elife-43481-supp2.docx]

**Supplementary file 2. Questions in the travel survey**

| - Residence - Place of work - Have you visited the forest in the previous 2 months? If yes, where did you go? - Have you been to another country in the previous 2 months? If yes, where did you go? - Did you frequently travel to another village/town/city for a purpose other than work? If yes, where did you go? - Other than this regular travel, have you been to another village/town/city in this country for a purpose other than work in the past 2 months? If yes, where did you go? |
| --- |
