# Supplementary material for: Mapping imported malaria in Bangladesh using parasite genetic and human mobility data
Source: eLife. 2019 Apr 2;8:e43481. doi: 10.7554/eLife.43481 (PMC6478433; doi:10.7554/eLife.43481)
Supplement: Supplementary file 3. [file elife-43481-supp3.docx]

**Supplementary file 3. Drug resistance markers**

|  | **Antimalarial** | **Gene** | **Amino Acid Positions** | **Wild Type Haplotype** |
| --- | --- | --- | --- | --- |
| K13 | artemisinin | *pfkelch13* | any mutation seen in BTB/POZ and propeller domains |  |
| DHFR | pyrimethamine | *pfdhfr* | 51, 59, 108, 164 | NCSI |
| DHPS | sulfadoxine | *pfdhps* | 436, 437, 540, 581, 613 | SAKAA |
| EXO | piperaquine | *exonuclease* | 415 | E |
| MDR‐1 | chloroquine, amodiaquine, lumefantrine, mefloquine | *pfmdr1* | 86, 184, 1246 | NYD |
| CRT | chloroquine | *pfcrt* | 72, 73, 74, 75, 76 | CVMNK |
| PGB (ART‐R genetic background) | artemisinin | *pfarps10* | 127 | VDNIT |
|  |  | *ferredoxin* | 193 |  |
|  |  | *pfcrt* | 326, 356 |  |
|  |  | *pfmdr2* | 484 |  |
